# Effect of Fungal and Fungal-Bacterial Tempe-Type Fermentation on the Bioactive Potential of Grass Pea Seeds and Flaxseed Oil Cake Mix

**DOI:** 10.1155/2024/5596798

**Published:** 2024-03-20

**Authors:** Bożena Stodolak, Maja Grabacka, Anna Starzyńska-Janiszewska, Robert Duliński

**Affiliations:** Department of Biotechnology and General Technology of Food, Faculty of Food Technology, University of Agriculture in Krakow, ul. Balicka 122, 30-149 Kraków, Poland

## Abstract

Tempe is an Indonesian food product traditionally obtained from soybeans through solid-state fermentation with *Rhizopus*. A variety of substrates can be processed into tempe in the presence of other microorganisms. In this study, grass pea seeds with the addition of flaxseed oil cake (20% w/w) were either fermented using individual mould strains—*Rhizopus oryzae*, *R. oligosporus*, and *Mucor indicus*—or cofermented with the moulds and *Lactiplantibacillus plantarum*. In the obtained products, the content of dietary fibre, B group vitamins, and the level of peptides and antioxidant potential in aqueous extracts were measured. Moreover, peptides, angiotensin I convertase inhibitor, and antioxidant activity were determined after *in vitro* digestion. The effect of digestates on the differentiation of enterocytes was also investigated. Fermentation generally resulted in a decrease in the dietary fibre, especially the insoluble fraction (30-50%). The product obtained with *R. oryzae* was the best source of riboflavin and thiamine among all tested. The fermentation process promoted the accumulation of water-soluble peptides and antioxidant compounds. After *in vitro* digestion, the largest amount of antioxidant and antiradical compounds was released from tempe obtained with *R. oryzae*. However, the enrichment of the products with antioxidants resulting from solid-state fermentation did not simply translate into an improvement in antioxidant potential after digestion. Generally, fermentation carried out in the presence of *L. plantarum* brought positive effects only in the case of *R. oligosporus* DSM 1964. Digestion products obtained from *R. oryzae* tempe had a positive effect on the viability of Caco-2 cells differentiated into enterocytes. Interestingly, a higher activity of differentiation markers (alkaline phosphatase and sucrase-isomaltase) was observed under the influence of digestate of *R. oryzae* and *L. plantarum* tempe.

## 1. Introduction

Tempe is a food product originating from Indonesia, traditionally obtained from soybeans by means of solid-state fermentation. A variety of other legume seeds, as well as cereals and pseudocereals, can also be utilized in this process. Tempe is gaining popularity and recognition, especially among the vegetarian and vegan population, as it can be considered a meat substitute [1].

Our research over the years has been focused on the use of tempe-type fermentation to process the seeds of grass pea, a valuable plant that is relatively little known but worth popularizing. As a result of the fermentation with *Rhizopus oligosporus*, protein digestibility was improved, and antinutritional compounds, including trypsin inhibitors, phytate, and flatulence causing oligosaccharides, were eliminated. The antioxidant capacity of the products was also enhanced [2, 3]. Flaxseed oil cake, a waste product after cold pressing oil, turned out to be a valuable addition to the grass pea seeds. The introduction of this component in the amount of 20-25% (w/w) to the fermentation substrate resulted in the enrichment of the obtained tempe in omega-3 fatty acids, as well as an improvement in the amino acid and phenolic profiles [4, 5].

Tempe-type fermentation is carried out with moulds of the *Rhizopus* genus, mainly *R. oligosporus*, but also *R. oryzae*, *R. microsporus*, and *R. stolonifer*. When the process is conducted in a traditional way with the use of starters, in addition to the indispensable *Rhizopus*, moulds of the *Mucor* genus, yeasts and lactic acid bacteria, may also play a role [1]. *Mucor indicus* can play a role in tempe fermentation as it is present in ragi (starters) and was isolated form traditionally obtained tempe products. This fungus, regarded as nonpathogenic, is used in Asia for production of other food or alcoholic drinks from starchy substrates [6, 7]. *Lactiplantibacillus plantarum* is one of the species often involved in the fermentation of vegetable-based foods, including legume seeds, by means of both submerged and solid-state fermentation [8]. Our research proved that fungal-bacterial cofermentation, with the participation of *L. plantarum* and *R. oligosporus*, resulted in obtaining more beneficial tempe products from grass pea seeds than fermentation with the mould alone [5]. An area worth further exploration is whether the bacterial influence could be considered a more general pattern for varying tempe-type mould strains.

The aim of the present study was to compare the bioactive potential of tempe products obtained from grass pea seeds with 20% (w/w) flaxseed oil cake addition, with the use of various moulds of the *Rhizopus* genus: *R. oligosporus*, *R. oryzae*, and *Mucor indicus*, and to determine whether the cofermentation process with the participation of *Lactiplantibacillus plantarum* would, as before, bring greater benefits than fermentation with the mould only. The study evaluated, among other things, the dietary fibre content and its fractional composition, the level of B group vitamins, and the antioxidant potential. The experiment also assessed how the antioxidant potential measured in water extracts would change after *in vitro* digestion in conditions simulating the human gastrointestinal tract, and whether the fermentation products could be a potential source of compounds that inhibit the angiotensin I converting enzyme activity. Their effects on cell differentiation markers from the Caco-2 line were also determined for selected tempe.

## 2. Materials and Methods

### 2.1. Inoculum

In the fermentation experiment, the following strains were used: *Mucor indicus* CBS 670.79 and *Rhizopus oryzae* CBS 372.63 (CBS-KNAW Collection, Westerdijk Institute), *Rhizopus oligosporus* DSM 1962 and NRRL 2710 (German Collection of Microorganism and Cell Cultures), and *R. oligosporus* ATTC 64063 (American Type Culture Collection). All strains were cultured on potato extract agar for 12 days, after which spores were collected using a sterile saline solution (8 g/L) supplemented with peptone (0.01 g/L) and Tween 80 (0.1 mL/L). To eliminate mycelial hyphae, the suspension underwent triple filtration (ø 11 *μ*m, Nylon Net Filters, Millipore, Cork, Ireland). Spore density was determined using the counting method in a Thoma chamber.

Following rehydration of a freeze-dried culture, *Lactiplantibacillus plantarum* DSM 20174 (from the German Collection of Microorganisms and Cell Cultures) was cultivated at 30°C for 24 hours in de Man, Rogosa, and Sharpe (MRS) broth obtained from BioMaxima S.A., Lublin, Poland. The bacterial cells underwent centrifugation followed by suspension in a sterile saline solution. Cell density was determined using the turbidimetric method with McFarland's standards.

### 2.2. Fermentation Substrate Preparation

The grass pea (*Lathyrus sativus* L.) seeds of the Krab cultivar were acquired from “Spójnia” Hodowla i Nasiennictwo Ogrodnicze (Nochowo, Poland). A portion of 120 g of seeds was boiled in tap water for 30 minutes. Following this, the seeds were soaked in tap water (four times the seed volume) for 18 hours at room temperature, manually dehulled, and subsequently boiled for 15 minutes in tap water (four times the seed volume) acidified to pH 4.5–5.0 with lactic acid. After draining the water, the seeds were cooled to room temperature.

For the flaxseed oil cake, 30 g (one portion) obtained from Przedsiębiorstwo Nasienne CENTRALA NASIENNA Sp. z o.o. (Sanok, Poland) were hydrated to 40% moisture content and acidified simultaneously to pH 4−5 using an appropriate quantity of 5% lactic acid. The mixture was then sterilized (121°C, 20 min) and cooled to room temperature.

A portion of grass pea seeds mixed with a portion of flaxseed oil cake (8 : 2 w/w, a mass of raw material before hydration processes) underwent lyophilization and were stored at 4°C until analysis.

### 2.3. Fermentation and Cofermentation Process

The substrate, prepared as given above, comprised grass pea seeds mixed with flaxseed oil cake in an 8 : 2 (w/w) ratio. It underwent inoculation with a spore suspension of the individual mould strain at a dose of 3·10^6^ spores per 150 g of raw material, or with a spore suspension of the individual mould strain and cells of *L. plantarum* at a concentration of 3·10^6^ cells per 150 g of raw material. The inoculated material was compacted into Petri dishes (ø 11 cm, with four dishes designated for each fermentation variation) and then incubated at 30°C. Fermentation was halted by steaming the resulting products for 10 minutes, either after 30 hours or, for the *R. oligosporus* NRRL 2710 variants, after 40 hours, once the mould mycelium uniformly covered the substrate. Following that, the fermented products underwent lyophilization and were then stored at 4°C for subsequent analysis.

**Figure 1:**
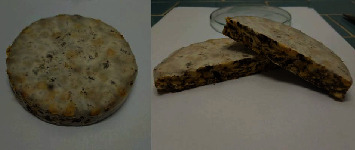
Tempe from grass pea and flaxseed oil cake (80 : 20, w:w) made with *R. oligosporus* NRRL 2710.

**Figure 2:**
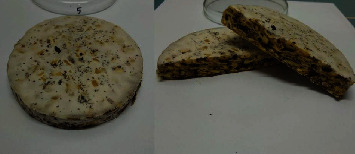
Tempe from grass pea and flaxseed oil cake (80 : 20, w:w) made with *R. oligosporus* DSM 1964.

Figures 1 and 2 show the representative examples of the obtained tempe products.

### 2.4. *Lactiplantibacillus plantarum* Count

To estimate the number of bacterial viable cells, 2 g of freshly obtained (nonsteamed) cofermented products were homogenized with a stomacher in 50 mL of sterile saline solution (0.85% NaCl) for 30 s. Next, a series of dilution from 10^−1^ to 10^−5^ was done. 50 *μ*L of each dilution was plated on the MRS agar supplemented with nystatin (100 IU/mL) and incubated for 48 h at 30°C.

### 2.5. pH Measurement

5 g of freshly obtained products after fermentation or cofermentation (before steaming) were homogenized with 20 mL of deionized water and mixed for 0.5 h. The pH measurements of the obtained slurry were repeated three times.

### 2.6. Preparation of Buffer Extracts

Extracts were prepared from the lyophilized material at a concentration of 1 g per 25 mL in a sodium-phosphate buffer (0.02 mol/L, pH 7.4). These extracts were utilized for the measurement of peptides, substances reacting with Folin-Ciocalteu reagent (FCRS), and ABTS^+·^ and ^·^OH scavenging activity.

### 2.7. Analytical Methods

Dry matter (DM) was determined using a moisture analyser (WPS 110S, Radwag, Radom, Poland) at 105°C. The glucosamine level in fungal fermented products (mg/g DM) was estimated by the colorimetric method as described by Starzyńska-Janiszewska et al. [9] after two-step acidic hydrolysis (10 mol/L HCl, 16 h at room temperature followed by 2 mol/L HCl, 2 h at 130°C). To calculate the fungal glucosamine, the amount of this compound present in the fermentation substrate was subtracted from the total estimated glucosamine level in the product. Dietary fibre, total, soluble, and insoluble (g/100 g DM) was measured according to the enzymatic-gravimetric method using Megazyme assay kit [9].

An estimation of thiamine as thiochrome and riboflavin levels (*μ*g/g DM) was performed according to Duliński and Starzyńska-Janiszewska [10] by reversed-phase high-performance liquid chromatography (HPLC) and fluorometric detection.

Peptide level was assessed using the o-phthalaldehyde method and expressed as mg/g DM based on a standard curve for reduced form of glutathione [9].

Antioxidant activity was measured as described by Stodolak et al. [5]. Briefly, for FCRS determination, 5 mL of appropriately diluted extracts were mixed with 0.25 mL of 1 mol/L Folin-Ciocalteu reagent and 0.5 mL of saturated Na_2_CO_3_ solution. Following a 15-minute incubation period, absorbance was measured at 700 nm against a blank, and the result was reported in milligrams of gallic acid per gram of dry matter (mg gallic acid/g DM). For hydroxyl radical scavenging activity (^·^OH-SA), various volumes of extracts were combined with potassium phosphate buffer (20 mmol/L, pH 7.4) to obtain a total volume of 1125 *μ*L. Subsequently, the mixture was supplemented with 40 *μ*L of 0.5 mmol/L FeCl_3_, 42 *μ*L of 2.4 mmol/L EDTA, 140 *μ*L of 0.02 mol/L deoxyribose, 10 *μ*L of 0.01 mol/L ascorbic acid, and 142 *μ*L of 1 mmol/L H_2_O_2_. Following an incubation period at 37°C for 1 hour, 1 mL of 1% (w/v) thiobarbituric acid (TBA) and 1 mL of 2.8% (w/v) trichloroacetic acid (TCA) were added. The sample was then incubated at 100°C for 20 minutes, and absorbance was measured at 532 nm against a blank. ^·^OH-SA was expressed as mg Trolox equivalents/g DM based on 50% free radical inhibition under the reaction conditions for each extract. For the ABTS radical scavenging activity (ABTS^+·^-SA), 200 *μ*L of properly diluted extracts were added to 2 mL of ABTS^+·^ solution with established absorbance of 0.7 ± 0.02 at 734 nm. Following a 7-minute incubation period at room temperature in the absence of light, absorbance was recorded at 734 nm using phosphate buffer as a reference. ABTS^+·^ scavenging activity was expressed as milligrams of Trolox equivalents per gram of dry matter (mg Trolox equivalents/g DM). For quencher-ABTS^+·^ assay (ABTS^+·^-QA), 10 mg of lyophilized material was combined with 30 mL of ABTS^+·^ solution (initial absorbance 0.7 ± 0.02 at 734 nm) and agitated at room temperature in the absence of light. Following a 7-minute incubation period, the absorbance of filtered samples was determined at 734 nm, with phosphate buffer used as a reference. The ABTS^+·^ scavenging activity was expressed in milligrams of Trolox equivalents per gram of dry matter (mg Trolox equivalents/g DM).

### 2.8. *In Vitro* Digestion Assay

*In vitro* digestion was performed according to international consensus method described by Minekus et al. [11] with some modification. 2 g of lyophilized material (in four replications) was firstly mixed with 1.6 mL of simulated salivary fluid, 0.01 mL 0.3 mol/L CaCl_2_, and 0.39 mL H_2_O and incubated for 2 minutes in a water bath at 37°C. After that, 2.4 mL of simulated gastric fluid (1.25× concentrated), 0.8 mL of 20000 U/mL pepsin (4750 U/mg, Sigma, Steinheim, Germany) dissolved in simulated gastric fluid, 0.002 mL 0.3 mol/L CaCl_2_, and 6 mol/L HCl and H_2_O in a final volume of 0.798 mL to adjust pH to 2 were introduced to the sample. The simulated digestion in the stomach was conducted in a water bath at 37°C with constant shaking for 2 h. Next, to neutralize the mixture to pH 7, 2 mol/L NaOH and H_2_O in a final volume of 1.384 mL were added. Then, the sample was mixed with 4.1 mL of simulated intestinal fluid (1.2× concentrated), 1.5 mL enzymatic solution in simulated intestinal fluid containing 10 mg of pancreatin (from porcine pancreas, 8xUSP specification, Sigma), 1 mL of 160 mmol/L bile acid solution, and 0.016 mL 0.3 mol/L CaCl_2_. The incubation simulated digestion in duodenum lasted for 2 h in a water bath at 37°C with constant shaking. Finally, the sample was centrifuged and stored at -80°C until analysed. A control sample—enzyme mix—containing only simulated digestion fluids, enzymes, and bile acid was also performed.

In digested samples, the peptide level and antioxidant activity (FCRS, ABTS^+·^-SA, ^·^OH-SA) were determined.

### 2.9. Angiotensin I Convertase (ACE) Inhibitor Activity

Determination of ACE inhibition was performed according to Udenigwe et al. [12]. As a source of ACE, acetone powder from rabbit lung (Sigma-Aldrich) was used. 0.3 g of powder was mixed with 7.5 mL borate sodium buffer (100 mmol/L, pH 8.3) and 0.27 mL 5% glycerol, shaken for 0.5 h, and centrifuged. 1 mL sample after digestion diluted with buffer (pH 8.3) to contain 0.5 mg of protein was mixed with 125 *μ*L of ACE extract and 180 *μ*L 1.6 mmol/L FAPGG (N-[3-(2-furyl(acryloyl]-Phe-Gly-Gly, Sigma-Aldrich). After 45 min incubation at 37°C, the reaction was stopped with 100 *μ*L 100 mmol/L EDTA. The absorbance was measured immediately at 340 nm (Asample). For each sample after digestion, a reference was made consisting of 1 mL of sample and 0.405 mL of buffer (Aref sample). Buffer was used instead of a sample after digestion in the blank experiment (Ablank). For blank probe, reference was also performed introducing EDTA before ACE extract (Aref blank).

Inhibitory activity (%) was calculated using the equation

(1) $$\begin{array}{c} \text{ACE inhibition}\left(\%\right)=100-\frac{\left(\left[\text{Aref blank}-\left(\text{Asample}-\text{Aref sample}\right)\right]\times 100\right)}{\left(\text{Aref blank}-\text{Ablank}\right)}. \end{array}$$

### 2.10. Caco-2 Cell Experiment

For experiments with Caco-2 cells, digestates of substrate and products after *R. oryzae* fermentation and *R. oryzae* and *L. plantarum* cofermentation were used.

### 2.11. Cell Culture

Cell culture experiments were performed on the Caco-2 human adenocarcinoma cell line (ATCC HTB-37), which was differentiated into enterocyte-like phenotype by the culture in multiwell plates with 0.4 *μ*m PET Transwell inserts. The differentiation procedure and the transepithelial electric resistance (TEER) measurements were carried out as described elsewhere [13]. The estimation of physiological doses of digestates for the cell culture treatment was carried out according to Hidalgo et al. [14] and Bottani et al. [15]. The calculation was based on assumption that an average serving of tempe product was 50 g, and the intestine surface covers roughly 200 m^2^. The volumes of digestates (*μ*L) per cm^2^ of cell culture area were 0.175 *μ*L/cm^2^, or in certain cases (where indicated), an average portion was set to 250 g, with the respective dose of 0.875 *μ*L/cm^2^.

The potential cytotoxicity of the digestates and their impact on enterocyte differentiation markers—alkaline phosphatase and sucrose-isomaltase enzymatic activities and the protein levels (determined by immunoblotting)—were performed exactly as described in Doniec et al. [13].

### 2.12. Statistical Analysis

For the analysis, four replications were made. The experiments with Caco-2 cells between two and five independent repetitions were made, each in tri- or tetraplicates. Data were processed using Statistica software version 13.3 (StatSoft, Inc., Tulsa, OK, USA) with a one-way analysis of variance and Fisher's post hoc test (*p* ≤ 0.05). For estimating the correlation between parameters, regression analyses were performed at *p* ≤ 0.05.

## 3. Results and Discussion

Dry matter losses indicate the metabolic activity of microorganisms during the fermentation process. In the present experiment, dry matter losses depended on both the type of microorganism and the fermentation method (Table 1). In the case of fermentation performed solely with fungi, the highest decrease in dry matter was determined in the product obtained with *R. oryzae* (15.4%). Among fungi used in this experiment, only *R. oryzae* is able to utilize oligosaccharides from the raffinose family present in legume seeds, including grass pea, as a carbon source [16–18], which could have resulted in better usage of substrate components, and a more intense metabolism. Changes in dry matter content, however, did not correlate with the level of fungal glucosamine determined in the products obtained with the exclusive use of moulds. Glucosamine content is considered one of the measures to estimate fungal biomass [19]. In the case of all *Rhizopus* strains used, a similar amount of glucosamine in tempe was measured at the end of the fermentation period (approx. 20 mg/g DM). Whereas, the product obtained with *Mucor indicus* was characterized by about 3-fold lower content of glucosamine. *M. indicus* is a dimorphic microorganism and, in aerobic conditions, may grow in the characteristic manner of moulds and in the typical manner of yeasts, simultaneously. The cell walls of yeast-like forms have been shown to have lower amounts of glucosamine and acetylglucosamine than that of filamentous forms [6].

In the process of cofermentation in the presence of *R. oligosporus*, the bacteria multiplied in the range of approx. 10,000 to 80,000 times their initial number. However, only in the case of *R. oligosporus* DSM 1964, after the cofermentation process, a significantly greater loss of dry matter was found, compared to the fermentation with the mould alone. LAB multiplied to a much lesser extent in the presence of *R. oryzae* (about 3,000 times). Perhaps, this strain, with a higher viability of spores compared to other moulds (approx. 73% versus 50-60%; unpublished data), very quickly dominated the substrate, competing with bacterial cells for available nutrients and thus inhibiting their multiplication.

The pH measured in the fermentation products ranged from 5.62 to 7.17 and depended more on the type of mould used than on the LAB inoculum. The lowest values were observed for *R. oryzae and R. oligosporus* NRRL 2710 with the addition of *L. plantarum*. It is known that *R. oryzae* is a producer of organic acids: lactic and fumaric [20]. The highest values, in turn, were measured for products obtained with *R. oligosporus* ATCC 64063 (pH 7.17), which may indicate advanced proteolysis, the final stage of which is the production of ammonia as a result of amino acid deamination [1]. The products of the metabolism of microorganisms affecting the final pH also shape the flavour and aroma characteristics of tempe.

### 3.1. B Vitamin Content

The level of B vitamins, riboflavin, and thiamine in the substrate was 1.22 and 2.3 *μ*g/g DM, respectively (Table 2). The fermentation performed solely with moulds enriched the material with vitamins of this group, with one exception (thiamine after fermentation with *R. oligosporus* NRRL 2710). The products contained from 2.5 to 11.5 times more riboflavin and up to twice as much thiamine, compared to the substrate. The richest source of both vitamins was found in tempe obtained with *R. oryzae*. This is in line with the findings of Omosebi and Otunola [21] who tested tempe products after fermentation with different *Rhizopus* species (*R. oryzae*, *R*. *oligosporus*, and *R. stolonifer*). The ability to synthesize B group vitamins during the tempe-type fermentation, with the exception of B12, has been proven for *Rhizopus* strains [22]. However, Wiesel et al. [16] observed that the strain of *R. oligosporus* MS5 was a much better producer of riboflavin than *R. oryzae* EN. Therefore, the ability to produce B vitamins may be a feature of strain rather than species. Enrichment of the inoculum in *L. plantarum* had a positive effect only on the level of riboflavin in *M. indicus* and *R. oligosporus* DSM 1964 variants (30% increase compared to the products obtained with mould solely). It should be emphasized that in the case of the substrate cofermented with the use of the mentioned fungal species, greater losses of dry matter were determined, than after fermentation with the moulds alone, i.e., the observed increase could simply be due to the concentration of the compound, calculated on the dry matter of the product. *L. plantarum* can synthesize both riboflavin and thiamine, but this depends largely on the ability of a particular strain [23, 24]. In this fermentation experiment, this was not the case—the producers turned out to be moulds, which is consistent with the work of Zhang et al. [25].

### 3.2. Dietary Fibre Content

The fermentation substrate had approximately 36 g/100 g DM of total fibre, of which 29% was soluble fraction (Table 2). Generally, two-thirds of the fibre found in foods is insoluble and one-third is soluble [26]. Polish cultivars of grass pea (the main component of the substrate) have over 33% total dietary fibre, of which less than 15% is soluble [27]. Flaxseed, in turn, contains 27 g/100 g of dietary fibre, of which one-third is soluble [28]. However, after defatting the seeds, the fibre level in flaxseed oil cake may exceed 50% [29]. The tempe obtained as a result of fungal fermentation and fungal-bacterial cofermentation was characterized by a lower content of total fibre than the initial substrate, 5% (*M. indicus*) to 35% (*R. oryzae* cocultivation), which was evidently due to the decrease in the insoluble fraction (by 7-54%). Consequently, the composition of the fibre was significantly modified, as the share of the soluble fraction increased from that found in the substrate by approx. 29% to even 50% (after fermentation with *R. oligosporus* DSM 1964 and cofermentation with *R. oryzae*). The benefits of soluble dietary fibre as a food component are connected with the cholesterol-reducing and blood-pressure-lowering effects, prevention of gastrointestinal problems, anticancerous activity, and an improvement in mineral bioavailability [30]. Moreover, in the functional food market, fortification in soluble fibre is considered advantageous as it provides viscosity and gel-forming ability [26]. Vig and Walia [31] and Lücke et al. [32] observed a reduction in fibre content, by 10 and 26%, respectively, after the fermentation of rapeseed oil cake with different *Rhizopus* species. It has been previously shown that, during tempe-type fermentation of soybean seeds, *R. oligosporus* produces enzymes that break down cell wall polysaccharides (endoglucanases, xylanases, and arabinases), the activity of which is highest after 20-30 hours of cultivation [33]. *M. indicus* and *R. oryzae* strains, in turn, are known as xylanase and cellulase producers, but the activity of the produced and secreted enzymes, capable of degrading cell wall components, depends on growth conditions [6, 20].

Tempe fermentation does not always result in a reduction in fibre content. Starzyńska-Janiszewska et al. [9, 34] observed an increase in total dietary fibre after the fermentation of wheat germ cake (14-28%) and quinoa seeds (27-59%). Interestingly, it was accompanied by an increase in the share of the soluble fraction. On the other hand, tempe-type fermentation of spelt did not bring significant changes in the level of fibre [35]. One of the components of fibre in fermentation products is fungal polysaccharides containing glucosamine, which accounted for 14% of the insoluble fraction, except variants obtained with *M. indicus* and *R. oligosporus* NRRL 2710. This data is consistent with the work of Starzyńska-Janiszewska et al. [9]. This is worth emphasizing because the mycelium of *Rhizopus*, a genus belonging to the *Zygomycetes*, in addition to chitin, also produces chitosan [36], a polysaccharide classified as a bioactive compound with numerous biological properties [37].

### 3.3. The Level of Peptides in Aqueous Extracts

The content of peptides found in aqueous extracts can give a general picture of the proteolytic degradation of substrate proteins. In addition, peptides present in tempe may constitute a pool of bioactive ingredients, showing, for example, antioxidative or antihypertensive capacity [38]. 11.33 mg of peptides per g of DM were determined in the substrate (Table 3). The fermentation process significantly enriched the products in peptides, from approx. 2-fold (*M. indicus*) to 10-fold (*R. oryzae*). Among the strains of *R. oligosporus* tested, the impact of NRRL 2710 was significantly lower than that of the other two strains (a 6-fold vs approx. 8-fold increase). The rise in the content of peptides in tempe compared to the substrate is the result of the proteolytic activity of microorganisms. One of the advantages of products obtained by means of tempe-type fermentation is a higher availability of nutrients, including proteins. Moulds typically used in this process have been shown to produce acidic (aspartyl) proteases with optimum pH 3 and 5.5, and alkaline, serine proteases with optimum at higher pH ≥ 7.0 [39, 40]. Differences in the level of peptides determined in fermentation products could result from varying metabolic activity of the moulds, as indicated by the losses of dry matter during the fermentation (Table 1). On the other hand, they might be a consequence of different distribution of activity of individual proteases during the process, as can be concluded from the research of Heskamp and Barz [39]. It was also shown that the highest proteolytic activity of *R. oryzae* was observed at pH 5.5 [41], which was the value determined in tempe at the end of the fermentation period (Table 1). Lactobacilli also show proteolytic activity, when applied in the process of proteolysis of plant proteins [42]. However, in this experiment, they did not contribute to an increase in the peptide content in fermentation products. The exception was tempe obtained with *R. oligosporus* DSM 1964, in the case of which a 20% higher level of peptides was found than in the analogous product fermented only with this mould.

### 3.4. Antioxidant Activity

The antioxidant activity of the tested materials (fermentation substrate and products) was determined on the basis of the level of compounds reacting with the Folin-Ciocalteu reagent, i.e., primarily phenols, and the ability to neutralize the synthetic ABTS radical and the hydroxyl radical generated during the analysis. The analyses were performed for aqueous extracts, as they were considered more relevant to *in vivo* activity, due to the fact that compounds soluble in water by their nature become more available during the digestive process.

#### 3.4.1. The Level of Compounds Reacting with Folin-Ciocalteu Reagent (FCRS)

Less than 2 mg/g DM of water-soluble phenols were measured in the substrate prepared for inoculation. In all products obtained by the fermentation, the content of phenols was higher than in the substrate (Table 3). The process conducted solely with moulds increased the phenol content from 1.15 times (*M. indicus*) to 3.3 times (*R. oligosporus* DSM 1964 and ATCC 64063). Both moulds and lactic acid bacteria synthesize enzymes whose activity contributes to increasing the solubility and activity of phenolic compounds [43, 44]. However, in this experiment, the addition of *L. plantarum* to the inoculum favoured the enrichment of the product in water-soluble phenols only in the case of cofermentation with *R. oligosporus* DSM 1964 (by 13%), which is consistent with our previous observations [5].

#### 3.4.2. Antiradical Activity

The neutralization activity of ABTS^+·^ by water-soluble compounds extracted from the substrate, calculated as Trolox equivalents, was 3.54 mg/g DM (Table 3). This is consistent with our previous study, in which 4.19 mg/g DM were determined for grass pea seeds with 20% flaxseed oil cake addition [5]. All the fermentation products were characterized by higher antiradical activity compared to the substrate. These changes were analogous to those observed in the case of phenols, because the increases in the activity of soluble compounds against ABTS^+·^ ranged from 1.3-fold (*M. indicus*) to 3.7-fold (*R. oryzae*). Moreover, the process of cofermentation improved ABTS^+·^-SA to a small extent only in the case of *R. oligosporus* DSM 1964 (by 15%, compared to fermentation with the mould alone).

**Figure 3:**
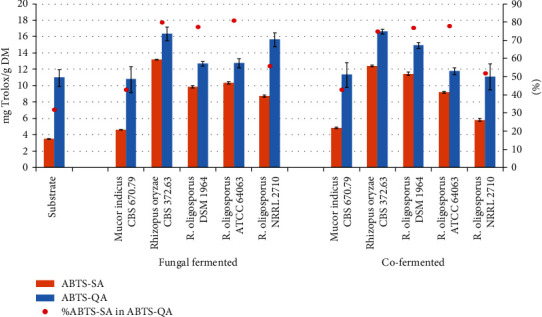
Effect of fermentation and cofermentation on ABTS^+•^scavenging activity. Data is shown as the mean ± SD.

The experiment also evaluated the total neutralization activity of the cation radical (ABTS^+·^-QA), capturing both the activity of soluble and insoluble compounds present in the tested material. The products obtained with *Rhizopus* strains and the cofermentation products obtained with *R. oryzae* and *R. oligosporus* DSM 1964 were characterized by a higher ABTS^+·^-QA than the substrate (by 15 to 50%). Moreover, fermentation contributed to an increase in the solubility of compounds capable of scavenging free radicals. This can be concluded from the comparison of the share of antiradical activity determined by the ABTS^+·^-SA method in the activity obtained by the ABTS^+·^-QA method (Figure 3). In the case of the substrate, this share was only 32%, indicating that a significant pool of compounds capable of neutralizing the free radical present in the material was insoluble in water. This is consistent with the observations of Wang et al. [45], who found that the antiradical activity of legume seeds dependent on the presence of insoluble phenolic compounds is higher than that resulting from the presence of soluble compounds. After the fermentation process, the share of ABTS^+·^-SA in ABTS^+·^-QA increased to 43% for products obtained with *M. indicus*, over 50% for products obtained with *R. oligosporus* NRRL 2710, and from 75 to 81% for other *Rhizopus* strains.

The ^·^OH neutralization activity of the aqueous extract from the substrate, expressed as Trolox equivalents, was 16.37 mg/g DM (Table 3). The fermentation process had a positive effect on this parameter. The ability to scavenge hydroxyl radical increased from less than 2-fold (fermentation with *M. indicus*) to 6-fold (*R. oryzae*). Among *R. oligosporus*, the weakest effects were observed for the NRRL 2710 strain. Again, only in the case of *R. oligosporus* DSM 1964, the addition of *L. plantarum* to the inoculum resulted in an enrichment of the product with compounds with the ability to scavenge ^·^OH (30% higher activity compared to the product of fermentation with the mould alone). As reported by Gan et al. [46], fermentation with *L. plantarum* can modify the antioxidant activity of legume seed products in a variety of ways, causing an increase in FCRS and, at the same time, reducing the antiradical activity.

In the presented experiment, the antiradical activity determined for aqueous extracts was highly correlated with both the level of peptides (*r*^2^ = 0.97 and 0.94, respectively, for scavenging ABTS^+·^ and ^·^OH) and FCRS (*r*^2^ = 0.74 and 0.88, respectively, for scavenging ABTS^+·^ and ^·^OH), indicating that the peptides and phenolic compounds determined by the method used were simultaneously able to scavenge free radicals or prevent their formation.

### 3.5. The Level of Peptides after *In Vitro* Digestion

After digestion simulating the human gastrointestinal tract, 143.2 mg of released peptides were measured in 1 g of DM substrate (Table 4). For most of the fermentation and cofermentation products, the amount of peptides determined after *in vitro* digestion was higher compared to the substrate, by 12% (*R. oligosporus* ATCC 64063) to a maximum of 40% (*M*.*indicus* + *L*.*plantarum*). Differences were not observed in the case of fermentation with *M. indicus* alone and both variants of fermentation with *R. oligosporus* NRRL 2710. It should be emphasized that the largest increases in the amount of peptides after *in vitro* digestion were observed in samples in which relatively few of those compounds were present before digestion (the substrate—by over 12 times, the products of fermentation and cofermentation with *M. indicus* and *R. oligosporus* NRRL 2710, by 6.6, 8, 2, and 5 times, respectively). For all other fermentation variants, the observed increases did not exceed twice the amount determined before digestion (Tables 3 and 4). The change in peptide levels after *in vitro* digestion observed in this experiment is consistent with that reported by Wang et al. [47]. The cited authors also observed a greater increase in the release of peptides from the material containing a smaller amount of them before digestion (over 3.5 times for the substrate, almost 2 times for the tempe).

### 3.6. Antioxidant Activity after *In Vitro* Digestion

#### 3.6.1. The Level of Compounds Reacting with Folin-Ciocalteu Reagent

*In vitro* digestion resulted in the release of 5.5 to a maximum of 6.84 mg of Folin-Ciocalteu reagent-reacting compounds from 1 g of material (Table 4). In the case of the substrate, and products obtained with *R. oligosporus* DSM 1964, these values were slightly lower than previously noted [5]. The observed differences were probably due to the change of the *in vitro* digestion method to the more recommended consensus method. The differences between the substrate and fermentation products reached a maximum of 24%, so they were also lower than in the cited paper (35%). The highest amount of FCRS was determined in the case of product obtained after fungal fermentation with *R. oryzae*. The effect of bacterial-fungal cofermentation on the availability of phenolic compounds was advantageous only in the case of *M. indicus*. Again, it is apparent that, as in the case of peptides, the digestion process significantly increased the availability of FCRS from the material less rich in these compounds. In the case of substrate, and after fermentation and cofermentation with *M. indicus* and *R. oligosporus* NRRL 2710, an increase by 180, 150, 170, 60, and 130% was recorded, respectively. Whereas, in the case of other products, the increases, if any, did not exceed 30% (Tables 3 and 4). This is consistent with the observations of Wang et al. [47], who found that although FCRS levels determined after *in vitro* digestion of tempe were higher compared to the substrate, the changes relative to the material before digestion were greater for the substrate.

#### 3.6.2. Antiradical Activity after *In Vitro* Digestion

ABTS^+·^-SA after *in vitro* digestion of the substrate was 24.66 mg Trolox/g DM, which is very close to the value reported in our previous study [5]. All fermentation products after *in vitro* digestion were characterized by a higher ability (from 6 to 40%) to scavenge this cation radical than the substrate (Table 4). The highest activity was measured for tempe obtained with *R. oryzae*. The cofermentation process resulted in the increase in the antiradical activity after digestion only in the case of *M. indicus* and *R. oligosporus* NRRL 2710. When analysing the changes of ABTS^+·^-SA after *in vitro* digestion in relation to the initial activity of water extracts, it should be reemphasized that the highest changes were observed for the substrate and the tempe obtained by means of fermentation and cofermentation with *M. indicus* and *R. oligosporus* NRRL 2710 (from 5 to 7 times). In addition, mg Trolox/g DM measured after digestion for all analysed samples (substrate and fermentation products) significantly exceeded the total activity of the material determined by the Quencher method. This means that the digestion process itself caused the formation of antiradical compounds.

After *in vitro* digestion, the activity to neutralize the hydroxyl radical was also measured and expressed as Trolox equivalents. This activity for the fermentation substrate was 72.32 mg/g DM, which is significantly higher than in our previous experiment [5].

The fermentation process, with minor exceptions (fermentation with *M. indicus* and cofermentation with *R. oligosporus* NRRL 2710), contributed to an increase in this activity after *in vitro* digestion (from less than 20% to a maximum of 54%). The highest scavenging activity of ^·^OH was shown by digested products of fermentation with *R. oryzae* and *R. oligosporus* NRRL 2710. Compared to fermentation with the moulds alone, the cofermentation process resulted in a significant increase in ^·^OH-SA in the case of *M. indicus* and a small but statistically insignificant increase in ^·^OH-SA in the case of *R. oligosporus* DSM 1964. Again, for this parameter, *in vitro* digestion contributed to the largest increases in hydroxyl radical neutralization activity for samples with the lowest initial activity (measured in aqueous extracts (Tables 3 and 4)).

It is noteworthy that the antiradical activity of ABTS^+·^-SA and ^·^OH-SA did not correlate with the level of FCRS or peptides, which may indicate that compounds released from the material during digestion, capable of scavenging free radicals, acted synergistically.

The results of this experiment clearly prove that the antioxidant activity of the material determined by simple *in vitro* tests can be significantly modified after the process of simulated digestion in the human digestive tract. Differences between samples before digestion do not translate directly to differences after digestion.

Wang et al. [47] also observed that after *in vitro* digestion, the antiradical activity was comparable for the substrate and the fermentation product, or slightly higher for tempe, as measured by means of the ABTS^+·^ test and the ^·^OH test, respectively. This was despite the fact that before digestion, the fermentation products showed a much higher ability to scavenge radicals than the substrate. Hence, we can repeat after Hidalgo et al. [14] that the enrichment of some material with antioxidants, in our case resulting from solid-state fermentation, does not translate into an improvement in antioxidant potential after *in vitro* digestion.

### 3.7. ACE Inhibitors

Most ACE inhibitors of natural origin are found in functional dairy products [48]. However, studies on the evaluation of the activity of ACE inhibitors derived from plant proteins in the process of their enzymatic hydrolysis are quite common. The source of inhibitors may also be, as in the case of milk, products generated as a result of fermentation of plant substrates [49].

Since ACE inhibitors, among others, can have a peptide structure, their activity may be altered after digestion in the human gastrointestinal tract. Hence, it is expedient to determine the activity of inhibitors potentially present in the gastrointestinal tract, which is why we decided to do so in the experiment described.

After *in vitro* digestion, some angiotensin I converting enzyme inhibitor activity was found (Table 4). The tempe obtained solely with *R. oligosporus* strains showed higher ACE inhibitory activity than the substrate. Moreover, the result of cofermentation was a slight (*R. oligosporus* ATCC 64063) or significant (*R. oligosporus* DSM 1964, *R. oryzae*) increase in ACE inhibition, as compared to the corresponding products of mould fermentation. This is consistent with the observations of Wu et al. [50], who found that the whole-grain oat cofermentation with *R. oryzae* and *L. plantarum* had a more beneficial effect on the ACE inhibitory potential than the fungal fermentation alone. It can therefore be concluded that the fermentation process facilitates the release of ACE inhibitory compounds during digestion. ACE inhibitory activity did not correlate with the total level of peptides determined after *in vitro* digestion. This indicates that both fermentation and cofermentation could have influenced the quality of peptides released from the substrate protein by microbial proteases during fermentation, and then by proteolytic enzymes applied during *in vitro* digestion. This is consistent with the research of Jakubczyk et al. [42], who studied the effect of the conditions and time of fermentation of pea seeds with *L. plantarum* on the release of peptides and the level of activity of ACE inhibitors after *in vitro* digestion.

Our observations are also in accordance with the results of Wang et al. [47]. These authors noted that differences in peptide levels between substrate and fermentation product are not a direct determinant of differences in ACE inhibitor activity levels. This may be due to the fact that the ACE-inhibiting activity of peptides is largely related to their molecular weight, as well as the amino acid composition, especially the C-terminal structure of these peptides [48]. The activity of angiotensin converting enzyme inhibitors was detected in soybean tempe and in lima bean tempe, obtained after fermentation with the commercial starter Raprima containing *R. oligosporus* cultures [51, 52]. Moreover, black common bean seeds fermented with *R. oligosporus* NRRL 2710 showed a significantly higher activity of ACE inhibitors after pancreatin digestion, than the fermentation substrate (IC_50_ 0.0321 vs 93.53 *μ*g/ml) [53], which proves the beneficial effect of this fermentation on blood pressure lowering potential. In contrast, Indrati et al. [54] reported that the type of inoculum used in the production of tempe (multistrain, monostrain) did not affect the potential activity of ACE inhibitors, indicating that this potential was more influenced by the type of substrate—legume seeds from which tempe was obtained. On the other hand, in the already cited work by Wang et al. [47], fermentation products had lower ACE inhibitory activity than the substrate after *in vitro* digestion.

### 3.8. The Impact of Digestates on the Epithelial Cell Differentiation

For the experiment with Caco-2 cells, the digestate of the fermentation substrate and tempe obtained with *R. oryzae* alone and *R. oryzae* and *L. plantarum* was taken, due to the fact that fermentation with this particular strain of mould appeared to be the most promising.

The influence of the bioactivity of the fermented products on the differentiation of Caco-2 cells into enterocyte-like phenotype was evaluated.

**Figure 4:**
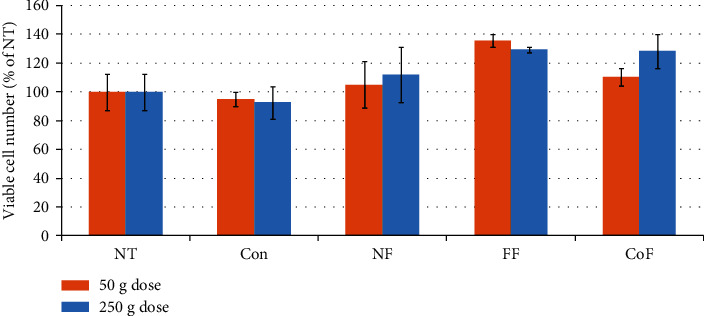
Effect of *in vitro* digestate on the viability of Caco-2 cells. Data is shown as the mean ± SD. NT: nontreated cells; Con: cell treated with *in vitro* digestion enzyme mix; FF: cells treated with *in vitro* digestion of product after *R. oryzae* fermentation; CoF: cells treated with *in vitro* digestion of product after *R. oryzae* and *L. plantarum* cofermentation. NF: cells treated with in vitro digestion of fermentation substrate.

**Figure 5:**
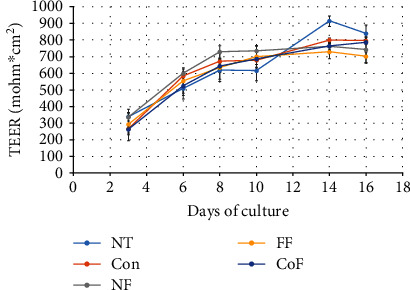
Transepithelial electric resistance (TEER) during Caco-2 cell culture. NT: nontreated cells; Con: cell treated with in vitro digestion enzyme mix; FF: cells treated with in vitro digestion of product after R. oryzae fermentation; CoF: cells treated with in vitro digestion of product after R. oryzae and L. plantarum cofermentation. NF: cells treated with in vitro digestion of fermentation substrate.

The MTS cytotoxicity test showed that the tempe digestates applied in either dose (a serving of 50 g or meal of 250 g) did not have any cytotoxic effect on Caco-2 cells (Figure 4). On the contrary, for the tempe obtained with *R. oryzae*, an increasing trend in the number of viable cells was observed (Figure 4). Caco-2 cells can be stimulated to differentiation when grown in a monolayer on the membrane inserts submerged in the cell culture medium. Differentiated Caco-2 cells form tight junctions and create nonpermeable barrier, which is characterized by high values of transepithelial electric resistance (TEER). Except for the *R. oryzae* fermented product, the tempe digestates did not affect the barrier function of Caco-2 cells, which reached high TEER values on the 16^th^ day of culture (Figure 5).

**Figure 6:**
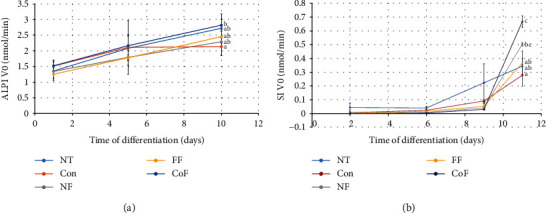
(a) Intestinal alkaline phosphatase and (b) sucrase-isomaltase activity during Caco-2 cell culture. Data is shown as the mean ± SD. NT: nontreated cells; Con: cell treated with *in vitro* digestion enzyme mix; FF: cells treated with *in vitro* digestion of product after *R. oryzae* fermentation; CoF: cells treated with *in vitro* digestion of product after *R. oryzae* and *L. plantarum* cofermentation. Different letters indicate significant differences (*p* ≤ 0.05). NF: cells treated with in vitro digestion of fermentation substrate.

**Figure 7:**
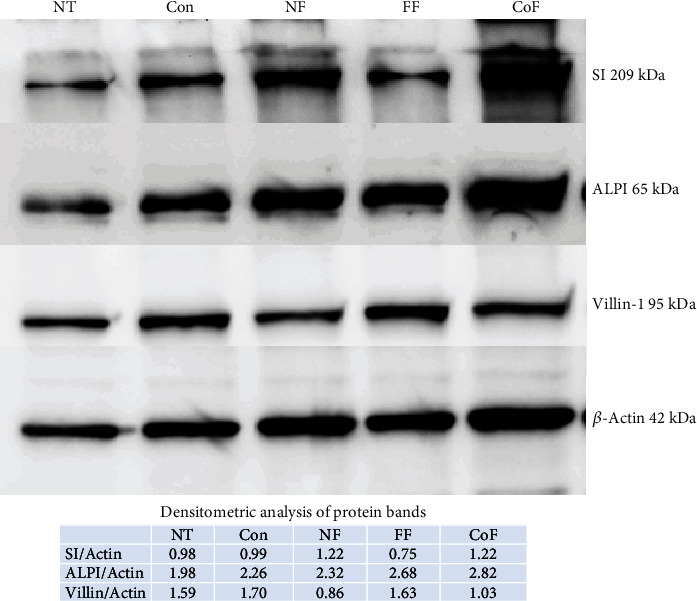
Immunoblotting analysis of alkaline phosphatase and sucrase-isomaltase proteins.

Next, the activity of two brush border enzymes, alkaline phosphatase (ALPI) and sucrase-isomaltase (SI), which are regarded as enterocyte differentiation markers, was measured. Interestingly, *R. oryzae* and *L. plantarum* fermented products significantly increased both ALPI and SI activities in the differentiated Caco-2 cells (Figures 6(a) and 6(b)). The high activities were accompanied by elevated protein levels of these enzymes detected by immunoblotting (Figure 7). The expression of villin 1, epithelial cytoskeleton filaments, was fairly constant in all the cultures (Figure 7).

## 4. Conclusions

The bioactive potential of tempe from grass pea with the addition of flaxseed oil cake depended on fungal species and *L. plantarum* activity. In terms of peptide content and antiradical potential (as measured by the ABTS cation radical test and hydroxyl radical neutralization), the most effective option was the fermentation process with *R. oryzae* strain. On the other hand, in the case of fermentation with *R. oligosporus* DSM 1964, the addition of *L. plantarum* in a dose equivalent to the amount of spores introduced, had more favourable results than fermentation with the mould alone. After *in vitro* digestion, *R. oryzae* tempe released the highest number of compounds reacting with the Folin-Ciocalteu reagent and scavenging the ABTS^+·^. However, the enrichment of the product with antioxidants by solid-state fermentation does not directly translate into the antiradical activity measured after *in vitro* digestion because the greatest absolute changes in relation to the material before digestion were observed for samples with the least amount of antioxidant compounds.

The presence of *R. oryzae* tempe digestion products favours the differentiation of Caco-2 cells into enterocytes. Interestingly, the digestate of tempe obtained after cofermentation with this mould increased both ALPI and SI activities in the differentiated Caco-2 cells. The effect of tempe on the viability and differentiation of Caco-2 cells is worth further investigation.

## Figures and Tables

**Table 1:** Dry mass loss, glucosamine content, pH value, and *Lactiplantibacillus plantarum* count after fermentation.

	Dry mass loss (%)	Glucosamine (mg/g DM)	*Lactiplantibacillus plantarum* count (fold of CFU/g FW)^∗^	pH
*Treatment kind*				
Fungal fermented				
*Mucor indicus* CBS 670.79	2.20 ± 0.45bc	6.67 ± 1.75a	—	6.84 ± 0.11c
*Rhizopus oryzae* CBS 372.63	15.43 ± 0.70g	20.37 ± 1.96b	—	5.83 ± 0.50a
*R. oligosporus* DSM 1964	3.83 ± 0.93d	20.14 ± 1.55b	—	6.66 ± 0.23bc
*R. oligosporus* ATCC 64063	2.15 ± 0.45bc	19.70 ± 1.80b	—	7.17 ± 0.21e
*R. oligosporus* NRRL 2710	1.61 ± 0.45a	21.75 ± 1.17b		6.54 ± 0.26b
Cofermented				
*Mucor indicus* CBS 670.79	2.88 ± 1.71cd	n.a.	5,746	6.88 ± 0.14cd
*Rhizopus oryzae* CBS 372.63	9.68 ± 0.55f	n.a.	3,034	5.62 ± 0.46a
*R. oligosporus* DSM 1964	5.36 ± 0.66e	n.a.	10,605	6.56 ± 0.22bc
*R. oligosporus* ATCC 64063	−0.79 ± 0.32a	n.a.	43,191	7.17 ± 0.10de
*R. oligosporus* NRRL 2710	0.17 ± 0.95a	n.a.	78,502	5.87 ± 0.66a

For dry mass loss, glucosamine content and pH value one-way of ANOVA were done. Data is shown as the mean ± SD. Mean values within a column followed by different letters differ significantly (*p* ≤ 0.05). ^∗^Increase of CFU/g of fresh weight of material inoculated with *L. plantarum* after fermentation.

**Table 2:** Dietary fibre and B group vitamin level in substrate and fermented products.

	Dietary fibre total (g/100 g DM)	Dietary fibre insoluble (g/100 g DM)	Dietary fibre soluble (g/100 g DM)	Riboflavin (*μ*g/g DM)	Thiamine (*μ*g/g DM)
Nonfermented	36.27 ± 0.08j	25.86 ± 0.16j	10.41 ± 0.08a	1.22 ± 0.14a	2.30 ± 0.13ab
Fungal fermented					
*Mucor indicus* CBS 670.79	34.51 ± 0.07h	23.99 ± 0.13i	10.53 ± 0.20a	4.14 ± 0.28d	4.75 ± 0.15f
*Rhizopus oryzae* CBS 372.63	25.71 ± 0.02c	13.76 ± 0.04c	11.95 ± 0.06b	14.02 ± 0.23j	4.89 ± 0.03f
*R. oligosporus* DSM 1964	29.07 ± 0.06e	14.47 ± 0.16e	14.60 ± 0.10f	8.52 ± 0.34fg	3.06 ± 0.15d
*R. oligosporus* ATCC 64063	25.08 ± 0.10b	13.30 ± 0.09b	11.78 ± 0.01b	9.61 ± 0.14h	3.62 ± 0.57e
*R. oligosporus* NRRL 2710	32.38 ± 0.14g	18.25 ± 0.11g	14.13 ± 0.03e	3.08 ± 0.10c	2.09 ± 0.09a
Cofermented					
*Mucor indicus* CBS 670.79	37.58 ± 0.08k	25.71 ± 0.15j	11.87 ± 0.07b	5.29 ± 0.54e	2.82 ± 0.18cd
*Rhizopus oryzae* CBS 372.63	23.83 ± 0.05a	11.85 ± 0.04a	11.98 ± 0.01b	8.26 ± 0.10f	2.53 ± 0.07bc
*R. oligosporus* DSM 1964	26.35 ± 0.09d	14.06 ± 0.04d	12.29 ± 0.14c	11.64 ± 0.41i	3.21 ± 0.21d
*R. oligosporus* ATCC 64063	30.43 ± 0.10f	16.84 ± 0.14f	13.59 ± 0.03d	8.85 ± 0.47g	3.88 ± 0.01e
*R. oligosporus* NRRL 2710	35.56 ± 0.21i	23.63 ± 0.10h	11.92 ± 0.11b	2.04 ± 0.05b	2.38 ± 0.09abc

One-way analysis of variance was applied. Data is shown as the mean ± SD. Mean values within a column followed by different letters differ significantly (*p* ≤ 0.05).

**Table 3:** Peptide level and antioxidant activity in buffer extracts.

	Peptide (mg/g DM)	FCRS (mg/g DM)	ABTS^+·^ SA (mg Tx/g DM)	ABTS^+·^ QA (mg Tx/g DM)	^·^OH-SA (mg Tx/ g DM)
Nonfermented	11.33 ± 0.80a	1.94 ± 0.04a	3.54 ± 0.03a	11.00 ± 1.02a	16.37 ± 1.18a
Fungal fermented					
*Mucor indicus* CBS 670.79	20.44 ± 1.17b	2.23 ± 0.03b	4.68 ± 0.04b	10.78 ± 1.60a	28.40 ± 1.92b
*Rhizopus oryzae* CBS 372.63	110.41 ± 4.72i	5.74 ± 0.08f	13.24 ± 0.05k	16.36 ± 0.86d	99.63 ± 6.62e
*R. oligosporus* DSM 1964	85.83 ± 2.77f	6.55 ± 0.14 g	9.87 ± 0.13g	12.71 ± 0.27b	74.93 ± 1.76d
*R. oligosporus* ATCC 64063	88.28 ± 4.21f	6.45 ± 0.11 g	10.37 ± 0.13h	12.78 ± 0.54b	74.47 ± 4.09d
*R. oligosporus* NRRL 2710	69.76 ± 4.21d	3.52 ± 0.07d	8.79 ± 0.13e	15.65 ± 0.84cd	43.16 ± 1.86c
Cofermented					
*Mucor indicus* CBS 670.79	24.67 ± 1.38b	2.30 ± 0.03b	4.88 ± 0.10c	11.33 ± 1.50a	28.90 ± 0.73b
*Rhizopus oryzae* CBS 372.63	95.41 ± 5.95 g	5.14 ± 0.12 e	12.48 ± 0.10 j	16.63 ± 0.27 d	78.48 ± 2.92 d
*R. oligosporus* DSM 1964	102.72 ± 2.38h	7.43 ± 0.12h	11.51 ± 0.21i	14.94 ± 0.37c	96.80 ± 4.35e
*R. oligosporus* ATCC 64063	80.52 ± 3.60e	5.85 ± 0.09f	9.23 ± 0.10f	11.76 ± 0.42ab	75.94 ± 3.88d
*R. oligosporus* NRRL 2710	29.63 ± 0.77c	2.56 ± 0.01c	5.85 ± 0.18d	11.11 ± 1.58a	24.50 ± 1.38b

One-way analysis of variance was applied. Data is shown as the mean ± SD. Mean values within a column followed by different letters differ significantly (*p* ≤ 0.05). FCRS: Folin-Ciocalteu reacting substances; ABTS^+·^-SA: ABTS^+·^-scavenging assay; ABTS^+·^-QA: ABTS^+·^ quencher assay, ^·^OH-SA: ^·^OH scavenging assay.

**Table 4:** Peptide level, antioxidant activity, and ACE inhibitor activity after *in vitro* digestion.

	Peptide (mg/g DM)	FCRS (mg/g DM)	ABTS^+·^ SA (mg Tx/g DM)	^·^OH-SA (mg Tx/g DM)	ACE inhibitor activity (%)
Nonfermented	143.20 ± 0.32a	5.50 ± 0.40a	24.66 ± 0.67a	72.32 ± 2.05a	11.18 ± 0.72b
Fungal fermented					
*Mucor indicus* CBS 670.79	134.95 ± 5.00a	5.55 ± 0.30a	26.13 ± 0.74b	66.88 ± 2.31a	5.21 ± 0.90a
*Rhizopus oryzae* CBS 372.63	189.71 ± 10.20d	6.84 ± 0.32d	34.30 ± 1.02g	111.93 ± 10.71d	11.50 ± 0.32b
*R. oligosporus* DSM 1964	166.31 ± 6.50bc	6.35 ± 0.15bc	30.90 ± 0.68e	85.11 ± 2.25b	19.30 ± 0.72e
*R. oligosporus* ATCC 64063	161.27 ± 7.80b	6.57 ± 0.25 d	26.52 ± 0.25b	94.37 ± 7.45c	16.79 ± 2.15d
*R. oligosporus* NRRL 2710	142.50 ± 10.73a	5.61 ± 0.18a	27.87 ± 0.76c	110.92 ± 9.21d	13.58 ± 0.32c
Cofermented					
*Mucor indicus* CBS 670.79	199.23 ± 4.47d	6.30 ± 0.35bc	32.65 ± 0.36f	90.05 ± 5.13bc	7.11 ± 0.36a
*Rhizopus oryzae* CBS 372.63	175.06 ± 7.14c	6.60 ± 0.41cd	29.28 ± 0.38d	97.93 ± 9.92c	14.38 ± 0.18c
*R. oligosporus* DSM 1964	174.68 ± 1.12 c	6.66 ± 0.45cd	28.45 ± 0.64cd	93.30 ± 7.45bc	24.66 ± 1.44f
*R. oligosporus* ATCC 64063	167.07 ± 6.52 bc	6.28 ± 0.28bc	28.84 ± 1.11cd	90.59 ± 1.43bc	18.44 ± 0.18de
*R. oligosporus* NRRL 2710	145.70 ± 12.45 a	5.91 ± 0.12 ab	31.91 ± 0.46ef	73.41 ± 1.45a	11.17 ± 0.77b

One-way analysis of variance was applied. Data is shown as the mean ± SD. Mean values within a column followed by different letters differ significantly (*p* ≤ 0.05). FCRS: Folin-Ciocalteu reacting substances; ABTS^+·^-SA: ABTS^+·^-scavenging assay; ^·^OH-SA: ^·^OH scavenging assay; ACE: angiotensin I converting enzyme.

## Data Availability

The data used to support the findings of this study can be made available from the corresponding author upon request.
